# Crystal structure and Hirshfeld surface analysis of *N*-(5-iodo-4-phenyl­thia­zol-2-yl)acetamide

**DOI:** 10.1107/S2056989019004791

**Published:** 2019-05-03

**Authors:** Angel D. Herrera-España, Jesús Aguilera-González, Gonzalo J. Mena-Rejón, Simón Hernández-Ortega, David Cáceres-Castillo

**Affiliations:** a Centro de Investigaciones Químicas, Instituto de Investigación en Ciencias Básicas y Aplicadas, Universidad Autónoma del Estado de Morelos, Av. Universidad 1001, Chamilpa, Cuernavaca 62209, Morelos, Mexico; b Facultad de Química, Universidad Autónoma de Yucatán, Calle 43 No. 613, Col. Inalámbrica, CP 97069, Mérida, Yucatán, Mexico; c Instituto de Química, Universidad Nacional Autónoma de México, Circuito Exterior, Ciudad Universitaria, 04510, Ciudad de México, Mexico

**Keywords:** crystal structure, 1,3-thia­zole, Hirshfeld Surface, hydrogen bonds, I⋯I and I⋯S inter­actions

## Abstract

Two crystallographically independent mol­ecules (*A* and *B*) are present in the asymmetric unit of the title compound, C_11_H_9_IN_2_OS, which differ mainly in the dihedral angle between the phenyl and thia­zole rings [38.94 (16) and 32.12 (15)°, respectively]. In the crystal, the mol­ecules form ⋯*A*⋯*B*⋯*A*⋯*B*⋯ chains along the [001] and [010] directions through moderate N—H⋯O hydrogen bonds and C—H⋯π inter­actions, respectively. The overall three-dimensional network is formed by I⋯I and I⋯S inter­actions. Hirshfeld surface analysis indicates that the most important contributions to the crystal packing are from H⋯C/C⋯H (26.2%), H⋯H (20.9%), H⋯I/I⋯H (19.4%) and H⋯O/O⋯H (6.8%) inter­actions.

## Abstract 2

The title compound crystallizes with two independent mol­ecules in the asymmetric unit. In the crystal, mol­ecules are linked by N—H⋯O hydrogen bonds, C—H⋯π, I⋯S and I⋯I inter­actions into a three-dimensional network.

## Chemical context   

The 1,3-thia­zole ring is a structural motif frequently found in the pharmaceutical field in anti­bacterial (Alam *et al.*, 2014 ▸), anti­fungal (Yu *et al.*, 2007 ▸) and anti­viral (Liu *et al.*, 2011 ▸) agents among others. In the chemotherapy of protozoal diseases, 5-bromo-2-amino­thia­zole derivatives have been investigated as privileged structures in biological tests against intestinal parasites such as *Giardia* (Mocelo-Castell *et al.*, 2015 ▸). Halo-1,3-thia­zole derivatives have proven to be suitable substrates in oxidative addition reactions in the presence of palladium (Wang *et al.*, 2015 ▸; Hämmerle *et al.*, 2010 ▸). The presence of halogens in the core of thia­zole derivatives opens the door to using them as suitable substrates for coupling reactions and to expand the therapeutic potential of a compound by improving the pharmaceutical properties. Transition-metal-catalysed reactions constitute one of the most important and attractive research areas in academia, as well as in the pharmaceutical and fine chemical industries (Zhao *et al.*, 2017 ▸; Jana *et al.*, 2011 ▸). Cross-coupling reactions usually require, in addition to a transition metal, that the electrophilic coupling partner possesses leaving groups such as Br^−^ or I^−^ among others. The development of suitable halo-1,3-thia­zole substrates for cross-coupling reactions allows us to report the crystal structure and the Hirshfeld surface analysis of *N*-(5-iodo-4-phenyl­thia­zol-2-yl)acetamide.

## Structural commentary   

**Figure 1:**
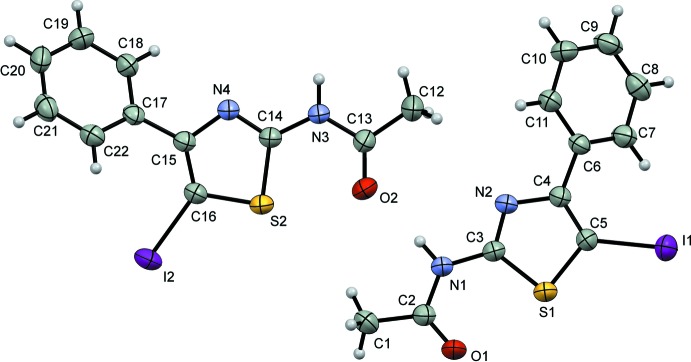
Mol­ecular structure of the two crystallographically independent mol­ecules in the asymmetric unit of the title compound, with the atom labelling. Displacement ellipsoids are drawn at the 50% probability level and H atoms are shown as spheres of arbitrary radius.

The title 2-aceto­amido­thia­zole derivative crystallizes in the monoclinic space group *P*2_1_/*c* with two crystallographically independent mol­ecules in the asymmetric unit (Fig. 1 ▸). The principal difference between these mol­ecules is the dihedral angle between the phenyl and thia­zole rings. In mol­ecule *A*, the thia­zole ring (S1/N2/C3–C5) makes a dihedral angle of 38.94 (16)° with the adjacent phenyl ring (C6–C11) while for mol­ecule *B* the dihedral angle between the S2/N4/C14–C16 and C17–C22 rings is 32.12 (15)°. Unlike the related compound 2-acetamido-4-*p*-tolyl-1,3-thia­zole (Lynch *et al.*, 2004 ▸) in which the mol­ecule is essentially flat, the presence of the iodine atom at C5 or C16 of the title compound induces rotation of the phenyl group attached to the thia­zole ring, as also observed in some bromine-substituted phenyl­thia­zole compounds (see the *Database survey*).
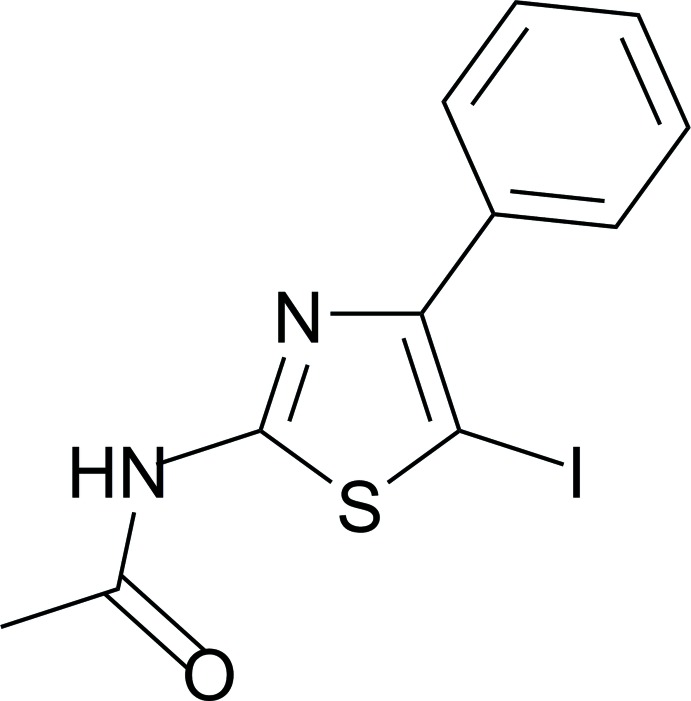


## Supra­molecular features   

**Figure 2:**
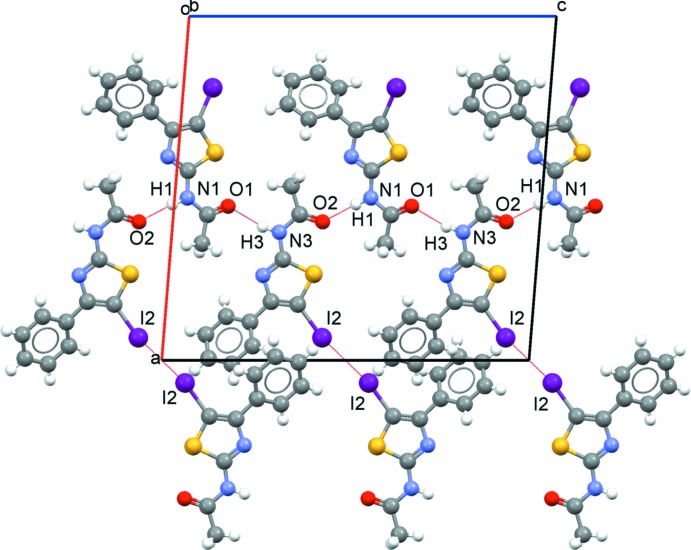
Part of the crystal structure of the title compound, showing the formation of hydrogen bonds and I⋯I contacts (red dashed lines) in the *ac* plane.

**Figure 3:**
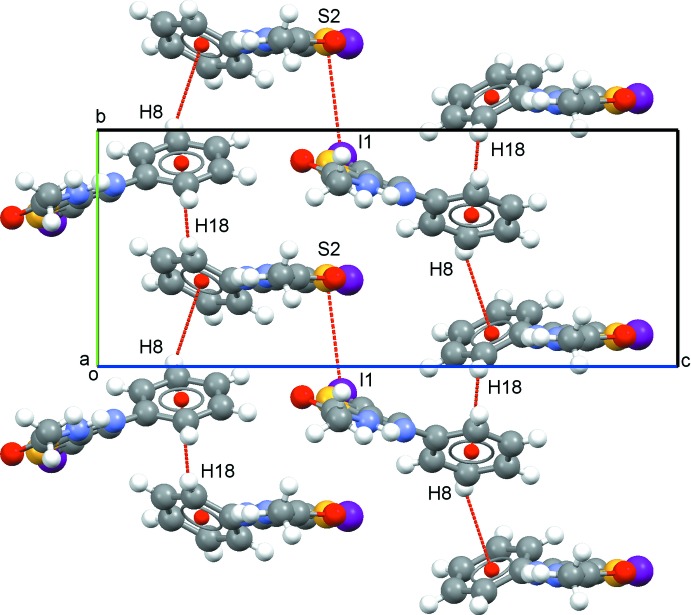
Packing viewed along the *a*-axis direction showing C–H⋯π and I⋯S inter­actions as red dashed lines.

In the crystal, mol­ecules are linked by N1—H1⋯O2 and N3—H3⋯O1 moderate hydrogen bonds *via* a *C*(4) synthon (Table 1 ▸, Fig. 2 ▸), forming chains along [001] in an ⋯*A*⋯*B*⋯*A*⋯*B*⋯ fashion. In the same way, the phenyl rings of mol­ecules *A* and *B* inter­act through C—H⋯π contacts along [010] and the resulting chains are further connected through I1⋯S2(1 − *x*, −*y*, 1 − *z*) contacts [3.7758 (9) Å] (Fig. 3 ▸). Additionally, adjacent *B* mol­ecules are linked by I2⋯I2(7 − *x*, 1 − *y*, 1 − *z*) contacts of type I [*θ*_1_ = *θ*_2_ = 146.91 (8)°] with a length of 3.8547 (5) Å.

## Hirshfeld surface analysis and two-dimensional fingerprints plots   

**Figure 4:**
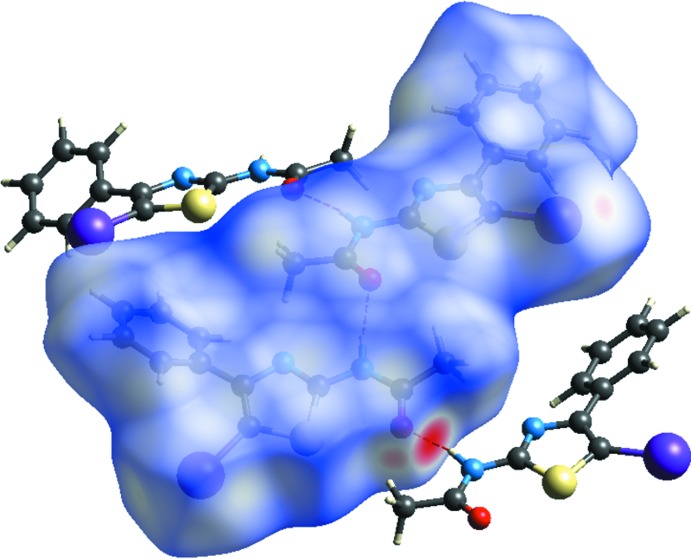
The three-dimensional Hirshfeld surface of the title compound mapped over *d*_norm_, showing the N—H⋯O hydrogen bonds.

**Figure 5:**
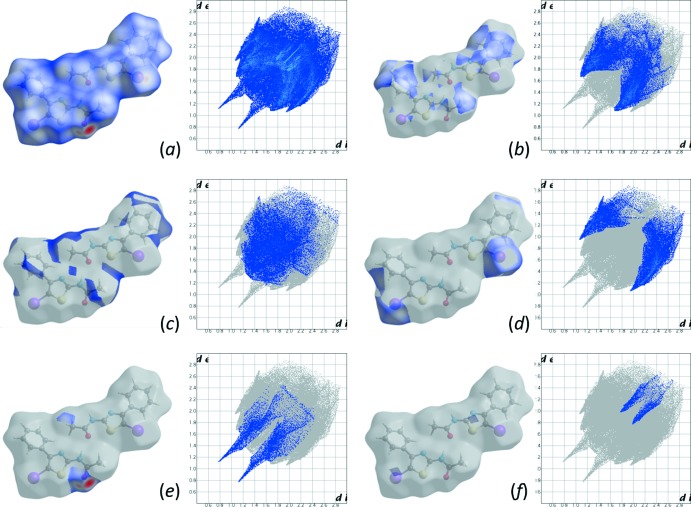
Two-dimensional fingerprint plots for the (*a*) all, (*b*) H⋯C/C⋯H (26.2%), (*c*) H⋯H (20.9%), (*d*) H⋯I/I⋯H (19.4%), (*e*) H⋯O/O⋯H (6.8%) and (*f*) I⋯S/S⋯I (2.2%) contacts in the title compound.

A Hirshfeld surface analysis was carried out using *Crystal Explorer17.5* (Turner *et al.*, 2017 ▸) in order to acquire a better understanding of the nature of the inter­molecular inter­actions in the title compound. The Hirshfeld surface was generated using a standard (high) surface resolution with the three-dimensional *d*_norm_ surface mapped over a fixed color scale of −0.5372 (red) to 1.3937 (blue) a.u. (Fig. 4 ▸). The intense red spots on the surface are due to the N—H⋯O hydrogen bonds, resulting from the inter­action of the amide group of the 2-aceto­amido­thia­zole derivative. The overall two-dimensional fingerprint plot for the title compound is shown in Fig. 5 ▸ *a*, and those delineated into H⋯C/C⋯H, H⋯H, H⋯I/I⋯H, H⋯O/O⋯H and I⋯S/S⋯I contacts are shown in Fig. 5 ▸ *b*–*f*. The major contribution to the crystal packing is from H⋯C/C⋯H inter­actions (26.2%). The pair of characteristic wings in this fingerprint plot corresponds to the C—H⋯π inter­actions between the phenyl groups (Fig. 5 ▸ *b*). The H⋯H and H⋯I/I⋯H contacts (Fig. 5 ▸ *c* and 5*d*) make similar contributions to the total Hirshfeld surface of 20.9 and 19.4%, respectively. The reciprocal H⋯O/O⋯H inter­actions (6.8%) are seen as sharp symmetrical spikes with tips at *d_e_* + *d_i_* ∼1.9 Å and arising from the N—H⋯O hydrogen bond (Fig. 5 ▸ *e*). Inter­molecular I⋯S/S⋯I (Fig. 5 ▸ *f*) and I⋯I inter­actions make smaller contributions to the Hirshfeld surface (2.2 and 1.1%, respectively).

## Database survey   

A search of the Cambridge Structural Database (CSD, Version 5.39, update of February 2018; Groom *et al.*, 2016 ▸) for a 1,3 thia­zole ring with a benzene ring and a halogen as substituents in positions 4 and 5, respectively, gave four entries for the organobromine compounds *N*-[5-bromo-4-(4-methyl­phen­yl)1,3-thia­zol-2-yl]-4-chloro­butanamide (CCDC 1443533; Ghabbour *et al.*, 2016 ▸), 5,5′-di­bromo-4,4′-bis­(penta­fluoro­phen­yl)-2,2′-bi-1,3-thia­zole (CCDC 889644; Siram *et al.*, 2013 ▸), 1-(5-bromo-4-phenyl-1,3-thia­zol-2-yl)pyrrolidin-2-one (CCDC 886962; Ghabbour, Kadi, *et al.*, 2012 ▸) and 5-bromo-4-(3,4-di­meth­oxy­phen­yl)-1,3-thia­zol-2-amine (CCDC 886876; Ghabbour, Chia, *et al.*, 2012 ▸). The dihedral angle between the thia­zole and benzene rings in these compounds are in the range 36.69 (11) to 60.83 (3)°, with exception of *N*-(5-bromo-4-(4-methyl­phen­yl)-1,3-thia­zol-2-yl)-4-chloro­butanamide. In this compound the dihedral angle is smaller [8.8 (3)°] as a result of an intra­molecular C—H⋯Br hydrogen bond. In the crystals of these compounds, only 5,5′-di­bromo-4,4′-bis(penta­fluoro­phen­yl)-2,2′-bi-1,3-thia­zole exhibits a type II halogen–halogen inter­action with a Br⋯Br distance of 3.6777 (3) Å and angles of 68.88 (5) and 174.77 (5)°.

## Synthesis and crystallization   

A mixture of *N*-(4-phenyl­thia­zol-2-yl) acetamide (0.5 mmol, 109 mg, 1 eq) and iodine (1 mmol, 127 mg, 2 eq) was placed in an open vessel containing a Teflon-coated stir bar. The mixture was dissolved in 3 mL of ethanol and the vessel was placed in the microwave cavity (CEM, Discover) and subjected to MW irradiation (150 W) for 60 min, at 363 K and a pressure of 2 psi. The reaction mixture was then cooled at room temperature and 5 mL of NH_4_OH were added. The obtained mixture was dissolved in ethyl acetate (50 mL) and washed with brine (3×). The organic layer was separated, dehydrated with Na_2_SO_4_, and evaporated in vacuo until dryness. The product was purified by flash column chromatography (silica gel, 2–25 µm) with a mixture of petrol–di­chloro­methane–acetone (5:3:2). The title compound was obtained as pale-yellow needles in 30% yield (52.2 mg, 0.15 mmol). A diluted solution of the compound was prepared in hexane and kept on a dry and dark place at room temperature. Crystals were obtained after one week of slow evaporation. Spectroscopic data: ^1^H NMR (400 MHz, CDCl_3_): 11.37 (*s*, 1H), 7.80 (*m*, 2H), 7.43 (*m*, 3H), 1.62 (*s*, 3H). ^13^C NMR (100 MHz, CDCl_3_): 168.8 (*s*), 163.6 (*s*), 151.4 (*s*), 134.5 (*s*), 129.0 (*d*), 128.9 (*d*), 128.7 (*d*), 62.4 (*s*) 21.9 (*c*).

## Refinement   

Crystal data, data collection and structure refinement details are summarized in Table 2 ▸. Hydrogen atoms bonded to C atoms were positioned geometrically and refined using a riding model: C—H = 0.95–1.00 Å with *U*_iso_(H) = 1.2*U*_eq_(C) or 1.5*U*_eq_(C-meth­yl).

## Supplementary Material

Crystal structure: contains datablock(s) I. DOI: 10.1107/S2056989019004791/lh5897sup1.cif

Structure factors: contains datablock(s) I. DOI: 10.1107/S2056989019004791/lh5897Isup2.hkl

Supporting information file. DOI: 10.1107/S2056989019004791/lh5897Isup3.cml

CCDC reference: 1908908

Additional supporting information:  crystallographic information; 3D view; checkCIF report

## Figures and Tables

**Table 1:** Hydrogen-bond geometry (Å, °) *Cg*2 and *Cg*4 are the centroids of the C6–C11 and C17–C22 rings, respectively.

*D*—H⋯*A*	*D*—H	H⋯*A*	*D*⋯*A*	*D*—H⋯*A*
N1—H1⋯O2	0.89 (3)	2.03 (3)	2.914 (3)	175 (3)
N3—H3⋯O1^i^	0.89 (2)	2.03 (2)	2.902 (3)	167 (2)
C8—H8⋯*Cg*4^ii^	0.93	2.94	3.655 (4)	134
C18—H18⋯*Cg*2^ii^	0.93	2.82	3.594 (4)	141

Symmetry codes: (i) 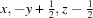; (ii) 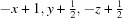.

**Table 2:** Experimental details

Crystal data	
Chemical formula	C_11_H_9_IN_2_OS
*M*_r_	344.16
Crystal system, space group	Monoclinic, *P*2_1_/*c*
Temperature (K)	298
*a*, *b*, *c* (Å)	17.4130 (6), 7.5325 (3), 18.5443 (6)
β (°)	94.567 (1)
*V* (Å^3^)	2424.61 (15)
*Z*	8
Radiation type	Mo *K*α
μ (mm^−1^)	2.79
Crystal size (mm)	0.30 × 0.27 × 0.09

Data collection	
Diffractometer	Bruker D8 Venture κ-geometry diffractometer 208039-01
Absorption correction	Multi-scan (*SADABS*; Krause *et al.*, 2015 ▸)
*T*_min_, *T*_max_	0.595, 0.745
No. of measured, independent and observed [*I* > 2σ(*I*)] reflections	21512, 4448, 3987
*R*_int_	0.020
(sin θ/λ)_max_ (Å^−1^)	0.604

Refinement	
*R*[*F* ^2^ > 2σ(*F* ^2^)], *wR*(*F* ^2^), *S*	0.029, 0.073, 1.12
No. of reflections	4448
No. of parameters	297
No. of restraints	2
H-atom treatment	H-atom parameters constrained
Δρ_max_, Δρ_min_ (e Å^−3^)	0.97, −0.49

Computer programs: *APEX3* and *SAINT* (Bruker, 2014 ▸), *SHELXS2014* (Bruker, 2014 ▸), *SHELXL2014* (Sheldrick, 2015 ▸), *Mercury* (Macrae *et al.*, 2006 ▸), *PLATON* (Spek, 2009 ▸) and *publCIF* (Westrip, 2010 ▸).

## supplementary crystallographic information

### Crystal data

**Table:**

C_11_H_9_IN_2_OS	*F*(000) = 1328
*M**_r_* = 344.16	*D*_x_ = 1.886 Mg m^−^^3^
Monoclinic, *P*2_1_/*c*	Mo *K*α radiation, λ = 0.71073 Å
*a* = 17.4130 (6) Å	Cell parameters from 9666 reflections
*b* = 7.5325 (3) Å	θ = 2.5–25.4°
*c* = 18.5443 (6) Å	µ = 2.79 mm^−^^1^
β = 94.567 (1)°	*T* = 298 K
*V* = 2424.61 (15) Å^3^	Prism, colourless
*Z* = 8	0.30 × 0.27 × 0.09 mm

### Data collection

**Table:**

Bruker D8 Venture κ-geometry diffractometer 208039-01	4448 independent reflections
Radiation source: micro-focus X-ray source	3987 reflections with *I* > 2σ(*I*)
Detector resolution: 52.0833 pixels mm^-1^	*R*_int_ = 0.020
ω–scans	θ_max_ = 25.4°, θ_min_ = 2.2°
Absorption correction: multi-scan (SADABS; Krause *et al.*, 2015)	*h* = −15→21
*T*_min_ = 0.595, *T*_max_ = 0.745	*k* = −9→9
21512 measured reflections	*l* = −21→22

### Refinement

**Table:**

Refinement on *F*^2^	2 restraints
Least-squares matrix: full	Hydrogen site location: mixed
*R*[*F*^2^ > 2σ(*F*^2^)] = 0.029	H-atom parameters constrained
*wR*(*F*^2^) = 0.073	*w* = 1/[σ^2^(*F*_o_^2^) + (0.0357*P*)^2^ + 2.3197*P*] where *P* = (*F*_o_^2^ + 2*F*_c_^2^)/3
*S* = 1.12	(Δ/σ)_max_ = 0.002
4448 reflections	Δρ_max_ = 0.97 e Å^−^^3^
297 parameters	Δρ_min_ = −0.49 e Å^−^^3^

### Special details

**Table:**

Geometry. All esds (except the esd in the dihedral angle between two l.s. planes) are estimated using the full covariance matrix. The cell esds are taken into account individually in the estimation of esds in distances, angles and torsion angles; correlations between esds in cell parameters are only used when they are defined by crystal symmetry. An approximate (isotropic) treatment of cell esds is used for estimating esds involving l.s. planes.

### Fractional atomic coordinates and isotropic or equivalent isotropic displacement parameters (Å^2^)

**Table:**

	*x*	*y*	*z*	*U*_iso_*/*U*_eq_	
I1	0.20804 (2)	0.11466 (3)	0.57958 (2)	0.05010 (9)	
I2	0.93426 (2)	0.36580 (3)	0.43149 (2)	0.05145 (9)	
S1	0.39704 (5)	0.14260 (11)	0.59979 (4)	0.04052 (18)	
S2	0.74959 (5)	0.37404 (11)	0.39534 (4)	0.03938 (18)	
O1	0.55222 (13)	0.1295 (3)	0.64721 (12)	0.0505 (6)	
O2	0.59773 (14)	0.3722 (3)	0.40790 (12)	0.0505 (6)	
N1	0.52822 (14)	0.2346 (4)	0.53438 (13)	0.0413 (6)	
H1	0.5467 (19)	0.278 (4)	0.4945 (12)	0.050*	
N2	0.40840 (14)	0.2698 (4)	0.47229 (13)	0.0388 (6)	
N3	0.62765 (15)	0.3834 (4)	0.29243 (13)	0.0408 (6)	
H3	0.612 (2)	0.377 (4)	0.2456 (7)	0.049*	
N4	0.75315 (14)	0.3745 (3)	0.25708 (13)	0.0372 (6)	
C1	0.66028 (18)	0.2219 (5)	0.58392 (19)	0.0510 (8)	
H1A	0.6829	0.2837	0.6256	0.076*	
H1B	0.6656	0.2923	0.5414	0.076*	
H1C	0.6860	0.1103	0.5792	0.076*	
C2	0.57687 (17)	0.1901 (4)	0.59246 (16)	0.0393 (7)	
C3	0.44875 (17)	0.2214 (4)	0.53026 (15)	0.0369 (6)	
C4	0.33023 (16)	0.2505 (4)	0.47953 (15)	0.0350 (6)	
C5	0.31384 (16)	0.1834 (4)	0.54453 (16)	0.0371 (6)	
C6	0.27744 (17)	0.3026 (4)	0.41671 (15)	0.0365 (6)	
C7	0.20880 (19)	0.3934 (4)	0.42351 (18)	0.0433 (7)	
H7	0.1925	0.4152	0.4692	0.052*	
C8	0.16480 (19)	0.4514 (5)	0.3630 (2)	0.0509 (8)	
H8	0.1190	0.5121	0.3681	0.061*	
C9	0.1884 (2)	0.4198 (5)	0.29482 (19)	0.0522 (9)	
H9	0.1591	0.4607	0.2541	0.063*	
C10	0.2554 (2)	0.3275 (5)	0.28749 (18)	0.0501 (8)	
H10	0.2710	0.3046	0.2416	0.060*	
C11	0.29986 (18)	0.2687 (4)	0.34786 (16)	0.0417 (7)	
H11	0.3450	0.2060	0.3423	0.050*	
C12	0.4936 (2)	0.3841 (7)	0.3156 (2)	0.0688 (12)	
H12A	0.4668	0.2859	0.3350	0.103*	
H12B	0.4900	0.3761	0.2638	0.103*	
H12C	0.4707	0.4934	0.3298	0.103*	
C13	0.57608 (18)	0.3794 (4)	0.34378 (17)	0.0413 (7)	
C14	0.70669 (17)	0.3778 (4)	0.30805 (15)	0.0354 (6)	
C15	0.82943 (17)	0.3668 (4)	0.28480 (15)	0.0338 (6)	
C16	0.83746 (17)	0.3663 (4)	0.35831 (16)	0.0363 (6)	
C17	0.88862 (17)	0.3640 (4)	0.23203 (16)	0.0340 (6)	
C18	0.87296 (17)	0.4513 (5)	0.16611 (16)	0.0418 (7)	
H18	0.8267	0.5121	0.1570	0.050*	
C19	0.92545 (19)	0.4480 (5)	0.11460 (18)	0.0494 (8)	
H19	0.9145	0.5069	0.0709	0.059*	
C20	0.99420 (19)	0.3583 (5)	0.1270 (2)	0.0495 (8)	
H20	1.0293	0.3562	0.0918	0.059*	
C21	1.01068 (18)	0.2721 (5)	0.19149 (19)	0.0477 (8)	
H21	1.0572	0.2119	0.2000	0.057*	
C22	0.95846 (18)	0.2741 (4)	0.24411 (17)	0.0404 (7)	
H22	0.9701	0.2152	0.2877	0.049*	

### Atomic displacement parameters (Å^2^)

**Table:**

	*U*^11^	*U*^22^	*U*^33^	*U*^12^	*U*^13^	*U*^23^
I1	0.03894 (13)	0.06520 (16)	0.04648 (14)	−0.01106 (9)	0.00551 (10)	0.00738 (10)
I2	0.04289 (14)	0.07225 (18)	0.03680 (13)	0.00079 (10)	−0.01192 (9)	−0.00143 (10)
S1	0.0370 (4)	0.0575 (5)	0.0268 (4)	−0.0013 (3)	0.0002 (3)	0.0065 (3)
S2	0.0389 (4)	0.0537 (5)	0.0252 (4)	−0.0033 (3)	0.0004 (3)	−0.0013 (3)
O1	0.0390 (12)	0.0830 (18)	0.0287 (11)	−0.0006 (11)	−0.0023 (9)	0.0113 (10)
O2	0.0479 (13)	0.0750 (17)	0.0293 (12)	−0.0054 (11)	0.0081 (10)	−0.0006 (10)
N1	0.0318 (13)	0.0654 (18)	0.0266 (13)	−0.0003 (12)	0.0010 (10)	0.0076 (12)
N2	0.0324 (13)	0.0572 (16)	0.0261 (12)	0.0005 (11)	−0.0020 (10)	0.0031 (11)
N3	0.0316 (13)	0.0656 (18)	0.0249 (12)	−0.0035 (11)	0.0015 (10)	−0.0014 (11)
N4	0.0309 (13)	0.0530 (16)	0.0275 (12)	−0.0010 (10)	0.0014 (10)	−0.0009 (10)
C1	0.0359 (17)	0.071 (2)	0.0444 (18)	0.0009 (16)	−0.0046 (14)	0.0087 (17)
C2	0.0363 (16)	0.0507 (18)	0.0307 (15)	0.0033 (14)	0.0006 (12)	−0.0016 (13)
C3	0.0341 (15)	0.0496 (18)	0.0269 (14)	−0.0006 (13)	0.0024 (12)	0.0015 (12)
C4	0.0315 (15)	0.0432 (16)	0.0300 (15)	0.0004 (12)	0.0004 (11)	−0.0019 (12)
C5	0.0308 (15)	0.0481 (17)	0.0323 (15)	−0.0034 (13)	0.0013 (12)	0.0022 (13)
C6	0.0325 (15)	0.0445 (17)	0.0313 (15)	−0.0051 (13)	−0.0044 (12)	0.0016 (13)
C7	0.0426 (18)	0.0506 (19)	0.0360 (16)	0.0042 (14)	−0.0015 (14)	0.0006 (13)
C8	0.0389 (18)	0.054 (2)	0.058 (2)	0.0072 (15)	−0.0064 (15)	0.0037 (17)
C9	0.050 (2)	0.063 (2)	0.0411 (19)	−0.0014 (17)	−0.0146 (15)	0.0079 (16)
C10	0.052 (2)	0.067 (2)	0.0300 (16)	−0.0039 (17)	−0.0016 (14)	0.0049 (15)
C11	0.0345 (16)	0.0541 (19)	0.0358 (16)	−0.0004 (14)	−0.0005 (13)	0.0053 (14)
C12	0.0346 (18)	0.129 (4)	0.044 (2)	−0.005 (2)	0.0073 (16)	−0.002 (2)
C13	0.0355 (16)	0.0538 (19)	0.0353 (17)	−0.0041 (13)	0.0059 (13)	−0.0032 (13)
C14	0.0340 (15)	0.0439 (17)	0.0279 (14)	−0.0019 (12)	0.0003 (12)	−0.0013 (12)
C15	0.0326 (15)	0.0380 (16)	0.0302 (15)	−0.0005 (12)	−0.0012 (12)	−0.0020 (11)
C16	0.0328 (15)	0.0438 (17)	0.0314 (15)	−0.0010 (12)	−0.0028 (12)	0.0004 (12)
C17	0.0306 (15)	0.0390 (16)	0.0316 (15)	−0.0027 (12)	−0.0017 (12)	−0.0064 (12)
C18	0.0328 (15)	0.0544 (19)	0.0376 (16)	0.0050 (14)	0.0001 (13)	0.0007 (14)
C19	0.0456 (19)	0.069 (2)	0.0339 (16)	0.0018 (17)	0.0043 (14)	0.0064 (16)
C20	0.0331 (17)	0.068 (2)	0.048 (2)	−0.0023 (15)	0.0115 (15)	−0.0061 (16)
C21	0.0327 (16)	0.056 (2)	0.055 (2)	0.0065 (14)	0.0008 (14)	−0.0093 (16)
C22	0.0375 (16)	0.0443 (18)	0.0382 (16)	0.0021 (13)	−0.0050 (13)	0.0007 (13)

### Geometric parameters (Å, º)

**Table:**

I1—C5	2.068 (3)	C7—C8	1.378 (5)
I2—C16	2.078 (3)	C7—H7	0.9300
S1—C5	1.734 (3)	C8—C9	1.381 (5)
S1—C3	1.735 (3)	C8—H8	0.9300
S2—C16	1.727 (3)	C9—C10	1.374 (5)
S2—C14	1.728 (3)	C9—H9	0.9300
O1—C2	1.222 (4)	C10—C11	1.382 (4)
O2—C13	1.220 (4)	C10—H10	0.9300
N1—C2	1.358 (4)	C11—H11	0.9300
N1—C3	1.383 (4)	C12—C13	1.490 (5)
N1—H1	0.890 (10)	C12—H12A	0.9600
N2—C3	1.289 (4)	C12—H12B	0.9600
N2—C4	1.386 (4)	C12—H12C	0.9600
N3—C13	1.360 (4)	C15—C16	1.359 (4)
N3—C14	1.384 (4)	C15—C17	1.477 (4)
N3—H3	0.891 (10)	C17—C22	1.394 (4)
N4—C14	1.292 (4)	C17—C18	1.396 (4)
N4—C15	1.387 (4)	C18—C19	1.374 (4)
C1—C2	1.493 (4)	C18—H18	0.9300
C1—H1A	0.9600	C19—C20	1.378 (5)
C1—H1B	0.9600	C19—H19	0.9300
C1—H1C	0.9600	C20—C21	1.371 (5)
C4—C5	1.358 (4)	C20—H20	0.9300
C4—C6	1.478 (4)	C21—C22	1.386 (4)
C6—C11	1.388 (4)	C21—H21	0.9300
C6—C7	1.392 (4)	C22—H22	0.9300
			
C5—S1—C3	87.64 (14)	C9—C10—H10	119.8
C16—S2—C14	87.64 (14)	C11—C10—H10	119.8
C2—N1—C3	125.7 (3)	C10—C11—C6	120.4 (3)
C2—N1—H1	120 (2)	C10—C11—H11	119.8
C3—N1—H1	114 (2)	C6—C11—H11	119.8
C3—N2—C4	111.3 (2)	C13—C12—H12A	109.5
C13—N3—C14	123.6 (3)	C13—C12—H12B	109.5
C13—N3—H3	121 (2)	H12A—C12—H12B	109.5
C14—N3—H3	115 (2)	C13—C12—H12C	109.5
C14—N4—C15	111.5 (2)	H12A—C12—H12C	109.5
C2—C1—H1A	109.5	H12B—C12—H12C	109.5
C2—C1—H1B	109.5	O2—C13—N3	120.9 (3)
H1A—C1—H1B	109.5	O2—C13—C12	123.9 (3)
C2—C1—H1C	109.5	N3—C13—C12	115.2 (3)
H1A—C1—H1C	109.5	N4—C14—N3	121.2 (3)
H1B—C1—H1C	109.5	N4—C14—S2	115.8 (2)
O1—C2—N1	120.9 (3)	N3—C14—S2	123.0 (2)
O1—C2—C1	123.9 (3)	C16—C15—N4	113.0 (3)
N1—C2—C1	115.3 (3)	C16—C15—C17	130.0 (3)
N2—C3—N1	120.1 (3)	N4—C15—C17	117.0 (2)
N2—C3—S1	115.8 (2)	C15—C16—S2	112.0 (2)
N1—C3—S1	124.0 (2)	C15—C16—I2	131.9 (2)
C5—C4—N2	113.7 (3)	S2—C16—I2	116.02 (16)
C5—C4—C6	129.6 (3)	C22—C17—C18	118.5 (3)
N2—C4—C6	116.7 (2)	C22—C17—C15	123.1 (3)
C4—C5—S1	111.5 (2)	C18—C17—C15	118.4 (3)
C4—C5—I1	128.9 (2)	C19—C18—C17	120.4 (3)
S1—C5—I1	119.55 (15)	C19—C18—H18	119.8
C11—C6—C7	118.7 (3)	C17—C18—H18	119.8
C11—C6—C4	118.2 (3)	C18—C19—C20	120.7 (3)
C7—C6—C4	122.9 (3)	C18—C19—H19	119.7
C8—C7—C6	120.5 (3)	C20—C19—H19	119.7
C8—C7—H7	119.7	C21—C20—C19	119.8 (3)
C6—C7—H7	119.7	C21—C20—H20	120.1
C7—C8—C9	120.2 (3)	C19—C20—H20	120.1
C7—C8—H8	119.9	C20—C21—C22	120.4 (3)
C9—C8—H8	119.9	C20—C21—H21	119.8
C10—C9—C8	119.7 (3)	C22—C21—H21	119.8
C10—C9—H9	120.2	C21—C22—C17	120.3 (3)
C8—C9—H9	120.2	C21—C22—H22	119.8
C9—C10—C11	120.5 (3)	C17—C22—H22	119.8
			
C3—N1—C2—O1	1.3 (5)	C14—N3—C13—O2	0.7 (5)
C3—N1—C2—C1	−178.3 (3)	C14—N3—C13—C12	−179.2 (3)
C4—N2—C3—N1	−178.5 (3)	C15—N4—C14—N3	−179.3 (3)
C4—N2—C3—S1	1.2 (4)	C15—N4—C14—S2	0.6 (3)
C2—N1—C3—N2	179.2 (3)	C13—N3—C14—N4	177.1 (3)
C2—N1—C3—S1	−0.5 (5)	C13—N3—C14—S2	−2.7 (4)
C5—S1—C3—N2	−0.9 (3)	C16—S2—C14—N4	−0.3 (2)
C5—S1—C3—N1	178.8 (3)	C16—S2—C14—N3	179.5 (3)
C3—N2—C4—C5	−0.9 (4)	C14—N4—C15—C16	−0.6 (4)
C3—N2—C4—C6	179.6 (3)	C14—N4—C15—C17	−179.5 (3)
N2—C4—C5—S1	0.3 (4)	N4—C15—C16—S2	0.4 (3)
C6—C4—C5—S1	179.6 (3)	C17—C15—C16—S2	179.1 (2)
N2—C4—C5—I1	−175.6 (2)	N4—C15—C16—I2	−177.5 (2)
C6—C4—C5—I1	3.7 (5)	C17—C15—C16—I2	1.3 (5)
C3—S1—C5—C4	0.3 (3)	C14—S2—C16—C15	0.0 (2)
C3—S1—C5—I1	176.6 (2)	C14—S2—C16—I2	178.15 (17)
C5—C4—C6—C11	−142.8 (3)	C16—C15—C17—C22	33.8 (5)
N2—C4—C6—C11	36.5 (4)	N4—C15—C17—C22	−147.5 (3)
C5—C4—C6—C7	41.2 (5)	C16—C15—C17—C18	−148.0 (3)
N2—C4—C6—C7	−139.5 (3)	N4—C15—C17—C18	30.7 (4)
C11—C6—C7—C8	−1.3 (5)	C22—C17—C18—C19	0.0 (5)
C4—C6—C7—C8	174.7 (3)	C15—C17—C18—C19	−178.2 (3)
C6—C7—C8—C9	0.1 (5)	C17—C18—C19—C20	0.2 (5)
C7—C8—C9—C10	1.0 (6)	C18—C19—C20—C21	−0.3 (6)
C8—C9—C10—C11	−1.0 (6)	C19—C20—C21—C22	0.3 (5)
C9—C10—C11—C6	−0.2 (5)	C20—C21—C22—C17	0.0 (5)
C7—C6—C11—C10	1.3 (5)	C18—C17—C22—C21	−0.1 (4)
C4—C6—C11—C10	−174.8 (3)	C15—C17—C22—C21	178.1 (3)

### Hydrogen-bond geometry (Å, º)

*Cg*2 and *Cg*4 are the centroids of the C6–C11 and C17–C22 rings, respectively.

**Table:**

*D*—H···*A*	*D*—H	H···*A*	*D*···*A*	*D*—H···*A*
N1—H1···O2	0.89 (3)	2.03 (3)	2.914 (3)	175 (3)
N3—H3···O1^i^	0.89 (2)	2.03 (2)	2.902 (3)	167 (2)
C8—H8···*Cg*4^ii^	0.93	2.94	3.655 (4)	134
C18—H18···*Cg*2^ii^	0.93	2.82	3.594 (4)	141

Symmetry codes: (i) *x*, −*y*+1/2, *z*−1/2; (ii) −*x*+1, *y*+1/2, −*z*+1/2.
